# Supplementary material for: Multicompartmental coacervate-based protocell by spontaneous droplet evaporation
Source: Nat Commun. 2024 Feb 6;15:1107. doi: 10.1038/s41467-024-45411-y (PMC10847435; doi:10.1038/s41467-024-45411-y)
Supplement: Supplementary file 1 — Supplementary Information [file 41467_2024_45411_MOESM1_ESM.pdf]

Supplementary Information

Multicompartmental coacervate-based protocell by spontaneous droplet evaporation

Cheng Qi[1], Xudong Ma[1], Qi Zeng[1], Zhangwei Huang[1], Shanshan Zhang[2], Xiaokang Deng[3], Tiantian Kong[2], Zhou Liu[3*]*

[1] Guangdong Provincial Key Laboratory of Micro/Nano Optomechatronics Engineering, College of Mechatronics and Control Engineering, Shenzhen University, Shenzhen, Guangdong 518060, China
[2] Department of Biomedical Engineering, School of Medicine, Shenzhen University, Shenzhen, Guangdong 518000, China
[3] College of Chemistry and Environmental Engineering, Shenzhen University, Shenzhen, Guangdong 518000, China

*Corresponding authors: ttkong@szu.edu.cn (T. Kong); zhouliu@szu.edu.cn (Z. Liu).

Supplementary Discussion

Supplementary Discussion 1 | Fluorescence recovery after photobleaching (FRAP) experiment of coacervate
Supplementary Discussion 2 | Partition of protein into PEO-rich and dextran-rich phases
Supplementary Discussion 3 | Effect of concentrations of PEO and dextran on formation of core-shell compartment
Supplementary Discussion 4 | Effects of temperature on formation of core-shell compartments
Supplementary Discussion 5 | Enhanced affinity of proteins-laden dextran-rich phase to substrate
Supplementary Discussion 6 | Evaporation of a sessile droplet on a clean substrate
Supplementary Discussion 7 | Morphology of ATPS droplets (without proteins) evaporating on organics-wetted and clean substrates resulting from different volume ratios of dextran and PEO
Supplementary Discussion 8 | Estimation of the relative importance of Marangoni and capillary effects
Supplementary Discussion 9 | Difference of interfacial tension at three-phase contact line and droplet edge
Supplementary Discussion 10 | Evaporation of a sessile droplet on a small pit
Supplementary Discussion 11 | Realization of partial coverage of sessile droplet by oil film

Supplementary References

Supplementary Discussion

Supplementary Discussion 1. Fluorescence recovery after photobleaching (FRAP) experiment of coacervate

To verify coacervation between lactoferrin and ovalbumin, we conduct a FRAP experiment using a sample prepared by mixing 10 wt % dextran, 5 wt % ovalbumin and 5 wt % lactoferrin. We label the proteins ovalbumin and lactoferrin with Rhodamine B-lactoferrin (red fluorescence) and FITC-lactoferrin (green fluorescence), respectively. At sufficiently high protein concentrations, the sample undergoes associative LLPS that forms droplets. To confirm the fluidic property of these droplets, FRAP experiment is performed by selecting a round area (enclosed by a white dashed circle) to execute photobleaching in the software NIS-Elements AR 5.41.00 and by setting the laser wavelengths of 561 nm and 488 nm to obtain Supplementary Figures 1A and 1B, respectively. Fluorescent images are captured at every 5 seconds, and are then analyzed by ImageJ to drawn Supplementary Figure 1C. The observed fluorescence recovery within 3 minutes provides evidence of liquid-like coacervate formation between lactoferrin and ovalbumin, substantiating their good fluidity within the droplets.

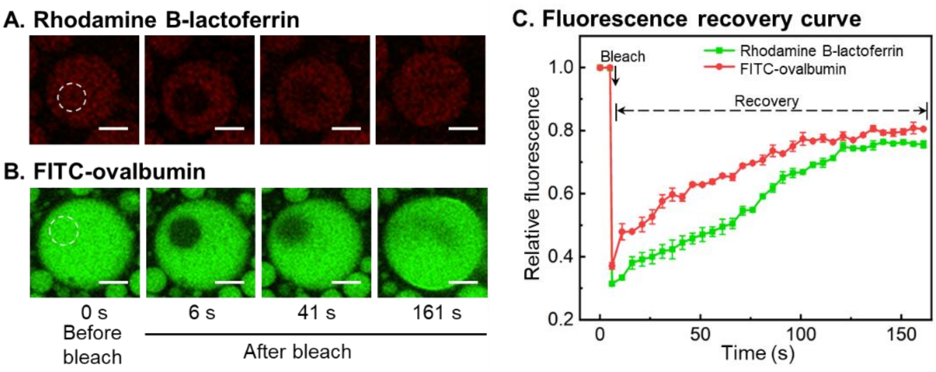

Supplementary Figure 1. FRAP experiments of lactoferrin/ovalbumin complex coacervate droplets with fluorescence-labelled Rhodamine B-lactoferrin and FITC-ovalbumin. Time-serial fluorescent images of a FRAP experiment of coacervates containing Rhodamine B-lactoferrin in (A) and FITC-ovalbumin in (B), and their corresponding fluorescence recovery curves in (C). In subfigures (A) and (B), Each experiment is independently repeated three times, and consistent results are obtained. The sample is prepared by mixing 10 wt % dextran, 5 wt % ovalbumin and 5 wt % lactoferrin. The proteins ovalbumin and lactoferrin are labelled with Rhodamine B-lactoferrin (red fluorescence) and FITC-lactoferrin (green fluorescence), respectively. Scale bars are 50 μm. Error bars represent standard deviation (SD), and $n = 3$ (independent experiments).

Supplementary Discussion 2. Partition of protein into PEO-rich and dextran-rich phases

Partition of proteins into PEO-rich and dextran-rich phases is obtained by Spark multimode microplate reader (TECAN). For the mixture solution containing 1.25 wt % PEO and 10.0 wt % dextran, after phase separation, the percentages of each protein (lactoferrin and ovalbumin) in PEO-rich and dextran-rich phases are listed in Supplementary Table 1. It indicated that both lactoferrin and ovalbumin are preferentially partitioned to the dextran-rich phase.

Supplementary Table 1. Percentages of proteins in PEO-rich and dextran rich phases. Bold denotes a larger percentage.

	Lactoferrin	Ovalbumin
PEO-rich phase	18.95%	22.42%
Dextran-rich phase	**81.05%**	**77.58%**

Supplementary Discussion 3. Effect of concentrations of PEO and dextran on formation of core-shell compartment

To systematically investigate the effects of concentrations of PEO and dextran on the formation of core-shell satellite compartments, we prepare four samples with different concentrations of PEO and dextran while concentrations of proteins are fixed at 0.5 wt % for both ovalbumin and lactoferrin. The concentrations of PEO and dextran in these four samples are: (A) 2.0 wt % dextran, 1.0 wt % PEO; (B) 1.5 wt % dextran, 0.5 wt % PEO; (C) 0.5 wt % dextran, 0 wt % PEO; (D) 0 wt % dextran, 0.5 wt % PEO. Rhodamine B (RB)-lactoferrin, RB-PEG, FITC-dextran, FITC-ovalbumin are added for fluorescent visualization. To perform the experiment, 300 μL silicone oil wets the petri dish first and 0.4 μL of the sample is pipetted onto the oil film. For the four samples, the corresponding patterns are shown in Supplementary Figure 2. In the absence of PEO or dextran, the core-shell compartment is not formed and only coacervates are found (Supplementary Figures 2C and 2D). Only in the case where both PEO and dextran are present, the core-shell compartments can be formed (Supplementary Figures 2A and 2B). Note that in the sample (A) the concentrations of PEO and dextran are sufficiently high that they already phase-separate before evaporation, which is distinguished from the sample (B). Although the core-shell structure can be formed for the sample (A), the coacervate-core is subtle (zoom-in in Supplementary Figure 2A(ii)).

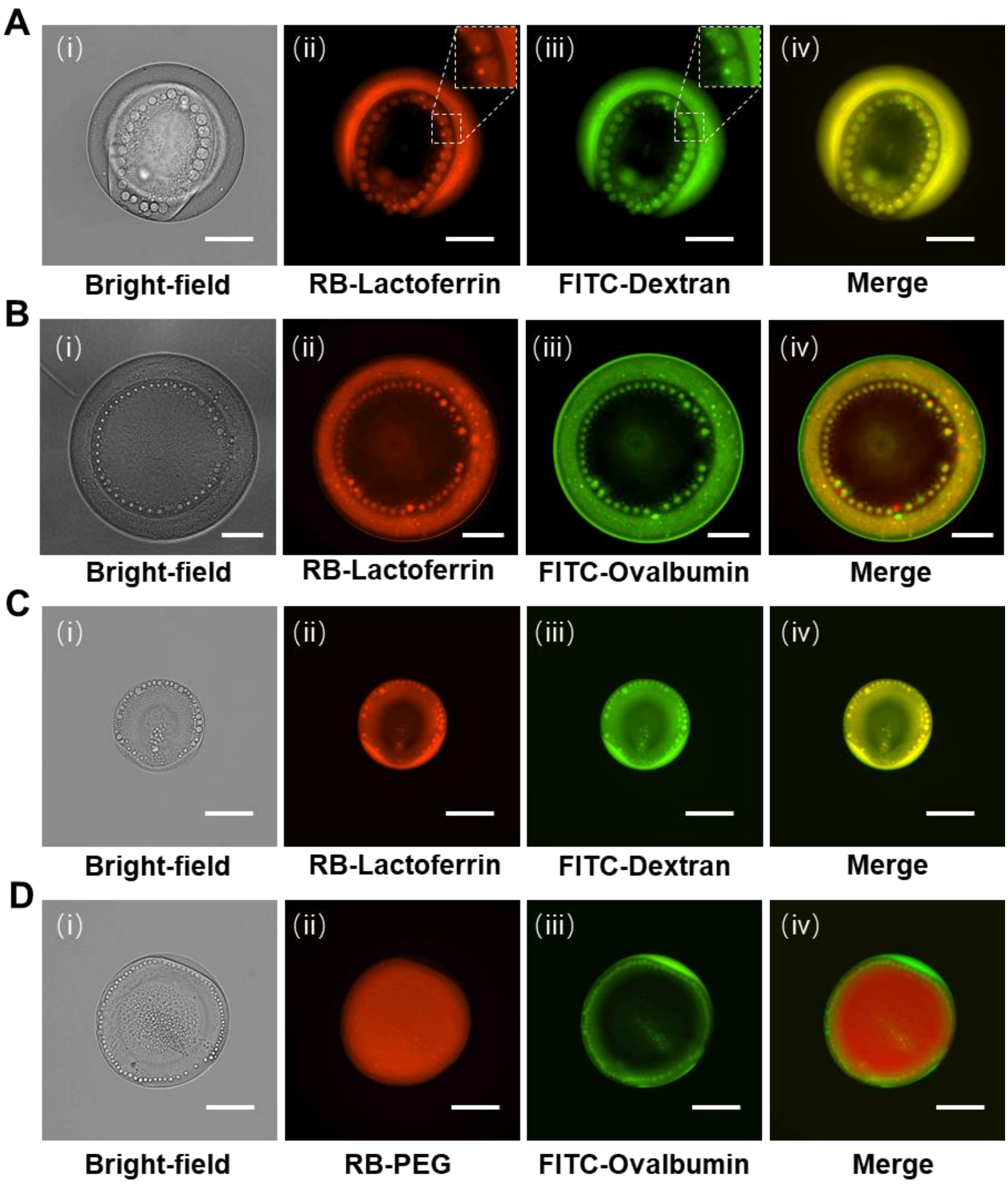

Supplementary Figure 2. Optical and fluorescent microscopic images showing patterns of evaporating sessile droplet consisting of different concentrations of PEO and dextran. Core-shell compartments are formed in (A) and (B), while are absent in (C) and (D). All scale bars are 200 μm. Each experiment is independently repeated three times, and consistent results are obtained.

Supplementary Discussion 4. Effects of temperature on formation of core-shell compartments

To investigate effects of temperature on formation of core-shell compartments during evaporation, we perform experiments at different temperatures ranging from 23 to 60 ºC. The liquid sample is prepared by mixing 1.5 wt % dextran, 0.5 wt % PEO, 0.5 wt % ovalbumin and 0.5 wt % lactoferrin. The temperature is controlled by Stage Top Incubator (TOKAI HIT, Japan). After a petri dish wetted by 300 μL oil is put inside the incubator, a customized temperature is set to heat the petri dish for 5 minutes. Then a droplet with a volume of approximately 0.4 μL of the sample is pipetted onto the petri dish and undergoes evaporation. For analysis, we define four stages and record the corresponding duration of each stage. The four stages are schematically depicted by Supplementary Figure 3A: Stage I, segregative LLPS is triggered and forms dextran-rich droplets dispersed in the PEO-rich continuous phase, and these droplets tend to fuse to minimize interfacial surface energy; Stage II, inside the fused dextran-rich droplet, associative LLPS occurs when coacervates start to appear and then to fuse; Stage III, the coacervates completely coalesce into a core such that a core-shell structure is formed and remains stable whereafter; Stage IV, the sessile droplet is completely dried. We find that the final core-shell structure is insensitive to temperature variations between 23 ºC and 60 ºC.

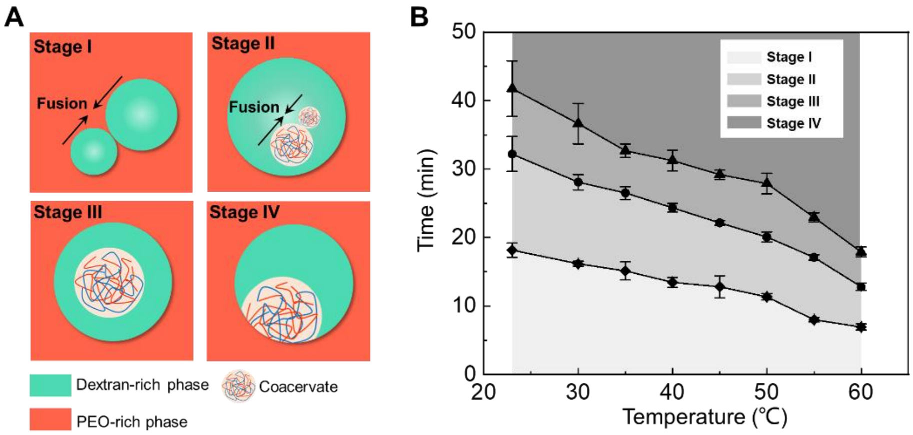

Supplementary Figure 3. Effects of temperature on formation of core-shell compartments. (A) Schematic diagrams of four stages regarding the formation of core-shell compartment of four stages during evaporation. (B) Duration of each stage as a function of temperature. Error bars represent SD, and $n = 3$ (independent experiments).

Supplementary Discussion 5. Enhanced affinity of proteins-laden dextran-rich phase to substrate

To study the affinity of protein-laden dextran-rich phase to the substrate, we conduct contact angle measurements using an inverted Motic AE2000 microscope. The substrate is first coated with trichloro (1H, 1H, 2H, 2H-tridecafluoron-octyl) silane (purity > 97% (GC), Aladdin) for 5 seconds, then is washed continuously with 95% ethanol and deionized water, and finally is put inside an oven (at the temperature about 70 °C) for one hour. For contact angle measurements, a droplet of 0.4 μL of the tested solution is then dropped onto the substrate. We measured four groups of solutions, the compositions of which are listed in Supplementary Table 2. The increase of proteins concentration in the dextran solution can lead to a decrease in the measured contact angle compared to that of a pure PEO solution, as summarized in Supplementary Figure 4. This suggests that the presence of sufficient proteins in the dextran-rich phase can effectively enhances its affinity to the substrate.

Supplementary Table 2. Compositions of the solutions prepared for contact angle measurement.

Group Number	Compositions
A	3.0 wt % dextran without protein
B	3.0 wt % dextran with 1.5 wt % proteins
C	3.0 wt % dextran with 5.0 wt % proteins
D	1.0 wt % PEO without protein

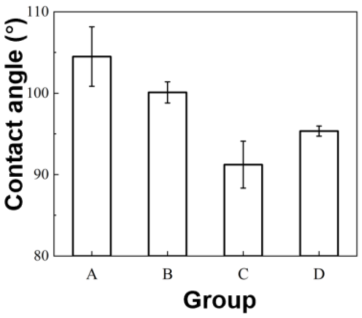

Supplementary Figure 4. Measurements of droplet contact angles on a substrate. Error bars represent SD, and $n = 3$ (independent experiments).

Supplementary Discussion 6. Evaporation of a sessile droplet on a clean substrate

To evaluate the role of the wetted oil film on the substate, we study the evaporation of a sessile droplet on a clean substrate. We prepare the liquid sample consisting of 1.5 wt % dextran, 0.5 wt % PEO, 0.5 wt % ovalbumin and 0.5 wt % lactoferrin. We drop 0.4 μL of the sample by micropipettor onto a petri dish for spontaneous evaporation. In the experiment, no oil phase is used to wet the petri dish aforehand. Rhodamine B (RB)-lactoferrin, RB-PEG, FITC-dextran, FITC-ovalbumin are added for fluorescent visualization. The pattern after evaporation is displayed in the below figure. Segregative LLPS between PEO and dextran occurs first to form a morphology of concentric circles (Supplementary Figure 5A). On the spherical interface between PEO- and dextran-rich phases, satellite coacervates are formed due to associative LLPS (Supplementary Figure 5B). However, core-shell compartments do not form in this case. By comparison of the patterns formed on both oil-wetted and unwetted substrates (as shown in Figure 2 and Supplementary Figure 5), we find that the oil phase indeed plays a crucial role in the formation and stabilization of core-shell coacervate droplets.

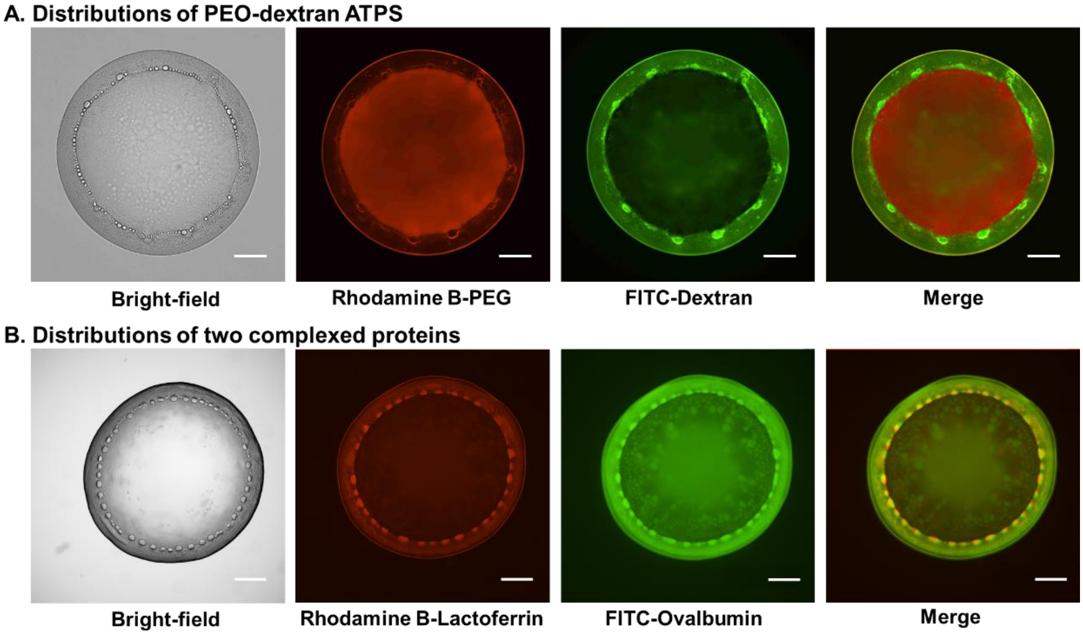

Supplementary Figure 5. Optical and confocal fluorescent microscopic images showing patterns formed during evaporation of a sessile droplet on a clean substrate and distributions of each component. (A) Confocal microscopic images show the locations of PEO-rich and dextran-rich phases. (B) Confocal microscopic images show the location of the coacervates (indicated by the overlapping region of FITC-ovalbumin and Rhodamine B-lactoferrin). All scale bars are 200 μm. Each experiment is independently repeated three times, and consistent results are obtained.

Supplementary Discussion 7. Morphology of ATPS droplets evaporating on organics-wetted and clean substrates at different volume ratios of dextran and PEO

We maintained the concentrations of dextran and PEO at 3.0 wt % and 1.0 wt %, respectively, throughout all experiments, while varying the volume ratio. We explored the influence of various dextran and PEO compositions on the droplet morphology during evaporation. Results are included in Supplementary Figure 6, where a circle of satellite microdroplets can form in the organics-wetted case regardless of the droplet composition.

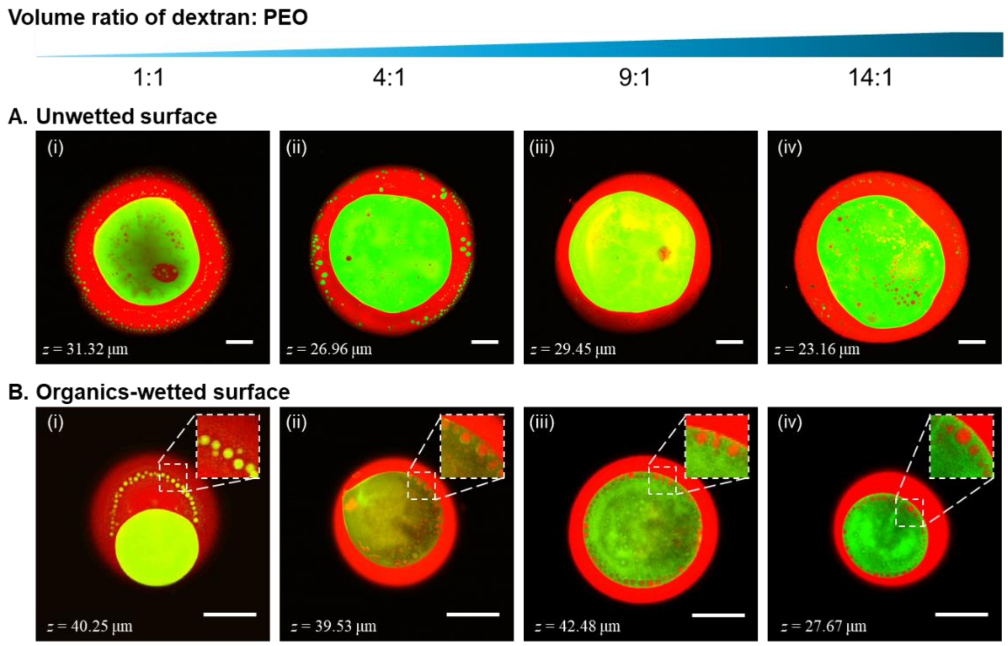

Supplementary Figure 6. Morphology of ATPS droplets evaporating on organics-wetted and clean substrates at different volume ratios from 1:1 to 14:1. For organics-wetted case, 300 μL silicone oil was used to wet the substrate *a priori*. At all samples, 1.0 wt % Pluronic F-68 was contained. All scale bars are 200 μm. Each experiment is independently repeated three times, and consistent results are obtained.

Supplementary Discussion 8. Estimation of the relative importance of Marangoni and capillary effects

An evaporating droplet assumed to be a nearly spherical cap geometry with a radius R and height h_0 was considered. As the droplet evaporated, the height of the liquid-air interface was denoted by $h(r, t)$, which was a function of the radial direction r and time t (Supplementary Figure 7).

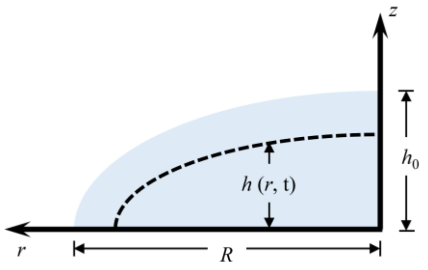

Supplementary Figure 7. Description of droplet geometry in the cylindrical coordinate (r, z). The initial radius and height of the sessile droplet is denoted by R and h_0. During its evaporation, its height h varies as a function of $h(r, t)$.

By assuming approximation ($h_0 \ll R$), the Navier-Stokes equation can be simplified to

$$\frac{\mathrm{d}p}{\mathrm{d}r} = \mu \frac{\partial^2 u}{\partial z^2} \quad \text{(S1)}$$

where p is the pressure, μ is the viscosity and u is the radial velocity. Given the boundary conditions of $\partial\gamma/\partial r \neq 0$ at $z = h$ and $u = 0$ at $z = 0$, by integrating Eq. (S1) with respect to z from gave the following velocity distribution[1,2]:

$$u(r, z, t) = \frac{1}{\mu}\frac{\mathrm{d}p}{\mathrm{d}r}\left[\frac{1}{2}z^2 - h(r, t)z\right] + \frac{z}{\mu}\frac{\partial\gamma}{\partial r}. \quad \text{(S2)}$$

The pressure is given by $p = -\gamma\nabla^2\tilde{h}$ where $\tilde{h}$ is the perturbation to the liquid-air interface caused by the internal flow. Thus, the interfacial velocity (i.e., the velocity at the liquid-air interface) is given by

$$u(r, t) = -\frac{h^2}{2\mu}\frac{\partial}{\partial r}\left(\gamma\nabla^2\tilde{h}\right) + \frac{h}{\mu}\frac{\partial\gamma}{\partial r}. \quad \text{(S3)}$$

The interfacial velocity has two components. The first and second terms in the right-hand side of Eq. (S3) are induced by Marangoni and capillary effects, respectively. The scales of these terms are at the orders of $(\gamma h_0^2\tilde{h})/(\mu R^3)$ and $(h_0\Delta\gamma)/(\mu R)$. Thus, the relative importance of the Marangoni and capillary effects can be expressed as a dimensionless parameter:

$$K = \left|\frac{\Delta\gamma}{\gamma}\frac{R^2}{h_0\tilde{h}}\right|. \quad \text{(S4)}$$

If $K \gg 1$, it indicates that the Marangoni effect is dominant over the capillary effect.

Supplementary Discussion 9. Difference of interfacial tension at three-phase contact line and droplet edge

Here, we describe the method to indirectly measure the interfacial tensions around the three-phase contact line and the droplet edge. We provide an example for a droplet containing 0.03125 wt % PEO, 3.50 wt % dextran and 1.0 wt % Pluronic F-68 (Supplementary Table 3). In addition, we add 0.015 mg/mL Rhodamine-PEG (polyethylene glycol, Mw = 40, 000, Aladdin) into the droplet. Then, we can establish a quantitative relationship between the fluorescent intensity and the concentration before evaporation (at the initial moment, $t = 0$ min). By using the software ImageJ, we measure the average gray values of the regions around the droplet edge (the area between two yellow dashed lines in Supplementary Figure 8A) and three-phase contact line (the area between two yellow dashed lines in Supplementary Figure 8B). We denote the corresponding gray values $\varepsilon_1 = 135.26$ (at the droplet edge) and $\varepsilon_2 = 147.41$ (at the contact line). After evaporating for 5 minutes, the average gray values change to $\varepsilon_1' = 108.93$ (the area between two yellow dashed lines in Supplementary Figure 8C) and $\varepsilon_2' = 213.09$ (the area between two yellow dashed lines in Supplementary Figure 8D), respectively.

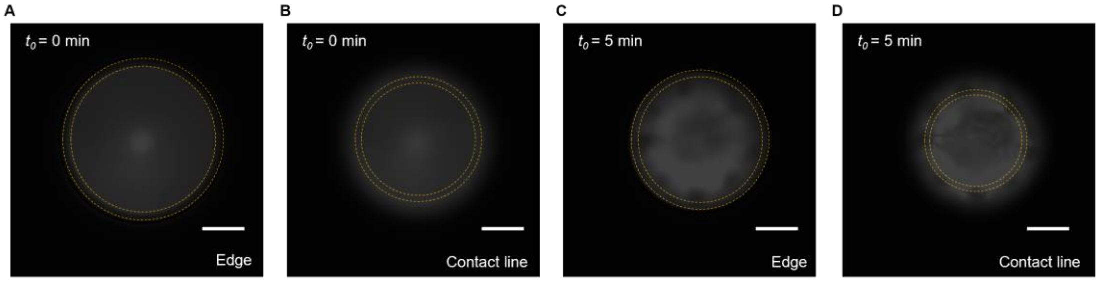

Supplementary Figure 8. Fluorescent images after graying in ImageJ of evaporating droplet at different times. Scale bars are 200 μm.

The gray value reflects the concentration of the substance inside the droplet. Then, it yields

$$\rho_1' = \frac{\varepsilon_1'}{\varepsilon_1}\rho_1 \text{ and } \rho_2' = \frac{\varepsilon_2'}{\varepsilon_2}\rho_2.$$

Initially, the concentrations of substance at the droplet edge and contact line are the same ($\rho_1 = \rho_2$) since the substance was uniformly distributed inside the droplet. Based on this, we can calculate the concentrations of PEO, dextran and Pluronic F-68 at $t = 5$ min (before phase separation occurred) (Supplementary Table 3). Using these calculations, we prepare the liquid samples containing 0.02517 wt % PEO, 2.81885 wt % dextran and 0.80539 wt % Pluronic F-68 and containing 0.04517 wt % PEO, 5.05945 wt % dextran and 1.44556 wt % Pluronic F-68, respectively. The interfacial tensions between the liquid samples and silicone oil are measured to be 14.70 mN/m and 20.70

mN/m, respectively, by using tensiometer (JYW-200B, Deka Precision Measuring Instrument (Shenzhen) Co., LTD).

Supplementary Table 3. Evaluation of substance concentrations after droplet evaporating for 5 minutes.

Concentration	0 min	5 min at Edge	5 min at Contact line
PEO	0.03125%	$\rho_1' = 0.025168\%$	$\rho_2' = 0.045174\%$
Dextran	3.50000%	$\rho_1' = 2.818854\%$	$\rho_2' = 5.059454\%$
F-68	1.00000%	$\rho_1' = 0.805387\%$	$\rho_2' = 1.445558\%$

Supplementary Discussion 10. Evaporation of a sessile droplet on a small pit

To demonstrate the effectiveness of the convective capillary flows in forming and maintaining core-shell compartments, we evaporate a sessile droplet on a small pit that can induce convective capillary flows. A solution of 1.0 wt % ethyl (2,4,6-trimethylbenzoyl) phenylphosphinate (purity 98%, Aladdin) as photo-initiator is mixed with trimethylolpropane ethoxylate triacrylate (ETPTA, average Mn ~ 428, Mackin). We place a 300 μL aliquot of the mixed solution onto the petri dish and refrigerate it at 4 °C, creating a small depression. After 0.5 hour in a cold water bath, we remove the dish from the refrigerator. We then drop 0.4 μL of deionized water onto the dish, creating a small pit as the water droplet impinges on the ETPTA-covered dish. The pit is solidified by exposing it to UV light (XP 104, AVENTK) at 365 nm for 40 seconds.

Next, we place a 0.4 μL sessile droplet on 300 μL oil containing 8.0 wt% RSN-0749 in the petri dish. The droplet consists of 3.0 wt% dextran and 1.0 wt% PEO in a volume ratio of 1:1. The proteins in the droplets have an initial concentration of 0.5 wt% before evaporation. During evaporation, we observe the formation of core-shell satellite droplets, as shown in the zoom-in region in Supplementary Figure 9.

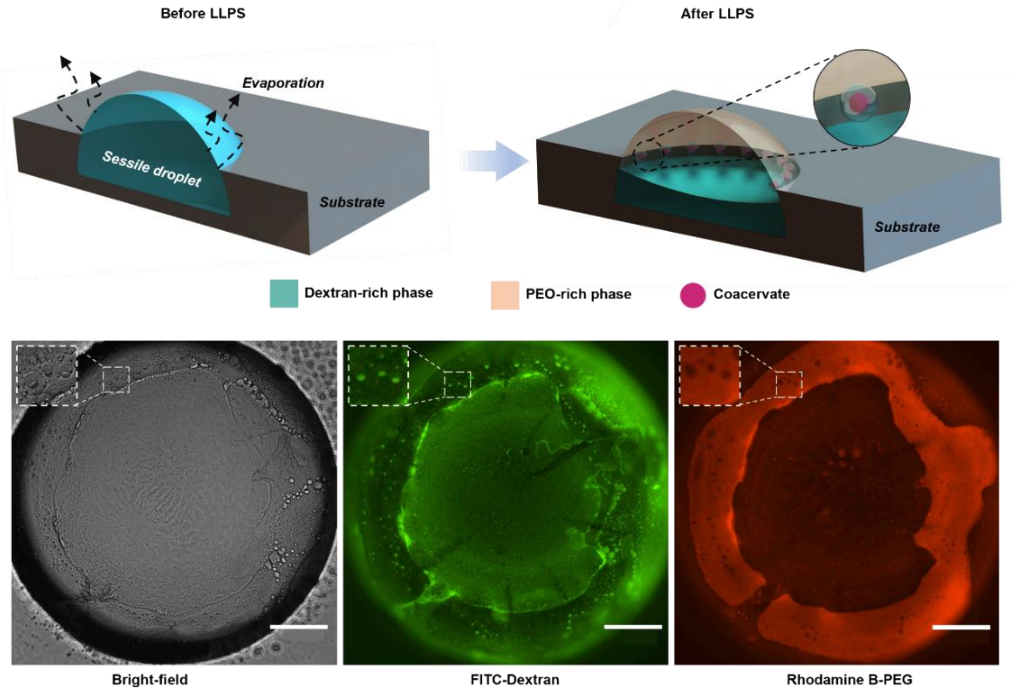

Supplementary Figure 9. Evaporation of droplet on a small pit. Core-shell satellite droplets are produced owing to multiple liquid-liquid phase separation (LLPS), which can be observed on the zoom-in region. Scale bars are 200 μm. Each experiment is independently repeated three times, and consistent results are obtained.

Supplementary Discussion 11. Realization of partial coverage of sessile droplet by oil film

The evaporation of sessile water droplets on an organics-wetted substrate reveals that a partial oil film coverage is crucial for forming a circle of stagnation points near the water-oil-air contact line. This partial coverage was experimentally achieved by either introducing water-soluble Pluronic F-68 into the water droplet or incorporating oil-soluble RSN-0749 into the oil phase.

To explore the effects of Pluronic F-68 and RSN-0749 on the coverage morphology, we pipetted a droplet onto a silicon oil-wetted substrate and observed the 3D reconstructed architecture with a confocal microscope. For fluorescent visualization, rhodamine B isothiocyanate-dextran (RBITC-dextran, red fluorescence) and perylene (blue fluorescence) were added to the the water droplet and oil film, respectively.

Three distinct scenarios were investigated: (A) a water droplet containing F-68 on a pure oil-wetted substrate; (B) a pure water droplet on an RSN-0749 laden oil-wetted substrate; (C) a pure water droplet on a pure oil-wetted substrate. The corresponding morphologies are shown in Supplementary Figure 10. In scenarios (A) and (B), the droplet exhibited partial coverage, while in scenario (C), it was completely engulfed by the oil film, as indicated by yellow arrows in Supplementary Figure 10C.

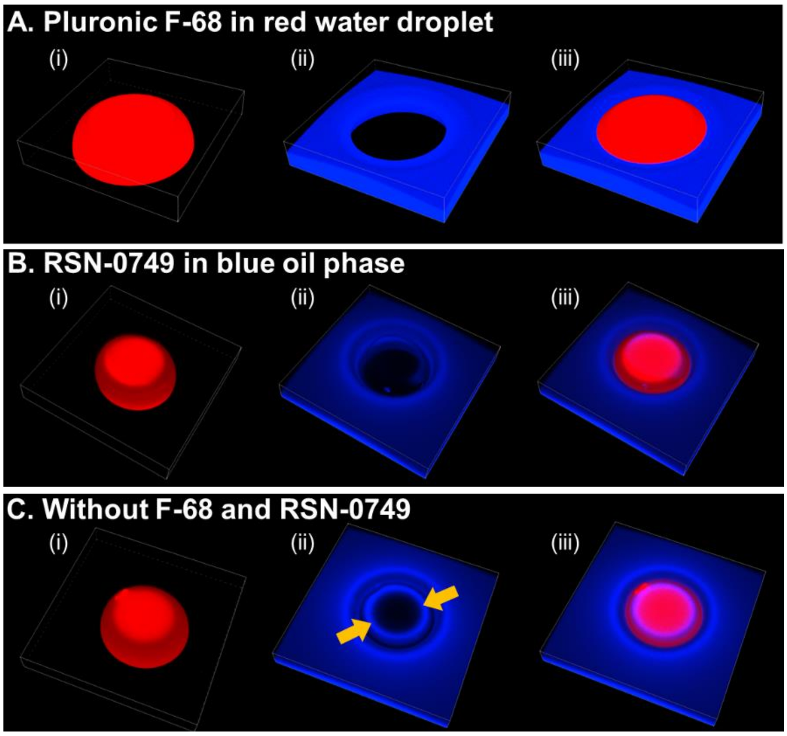

Supplementary Figure 10. Coverage morphology of a water sessile droplet on a silicon oil wetted substrate. Pluronic F-68 (1.0 wt %) and RSN-0749 (8.0 wt %) are added to the water droplet

(A) and oil phase (B), respectively. There are no additives in (C) which acts as a negative control. The water and oil phases are fluorescently labeled with RBITC-dextran (red) and perylene (blue), respectively. In (C), the oil film climbs onto the top of the sessile droplet, as denoted by yellow arrows.

Both Pluronic F-68 and RSN-0749 can reduce the coverage of oil film. Comparative analysis of Supplementary Figures 10A and 10B suggests that Pluronic F-68 is more effective, evidenced by minimal oil wetting on the droplet's surface in Supplementary Figure 10B(ii). However, Pluronic F-68 could interfere with proteins in LLPS droplet during evaporation. Therefore, for experiments involving proteins within the droplet, as shown in Figures 2 and 5, RSN-0749 was used. In other experiments (Figures 3 and 4), we used Pluronic F-68.

Without either RSN-0749 or Pluronic F-68, the droplet becomes fully covered by the oil film, due to the lower surface tension of silicone oil compared to water. In this scenario, the stagnation ring is absent and immobilization effect disappears; thus, microdroplets tended to coalesce into a bulk phase rather than forming a circle. Eventually, a Janus-like structure of droplet is produced (Supplementary Figure 11).

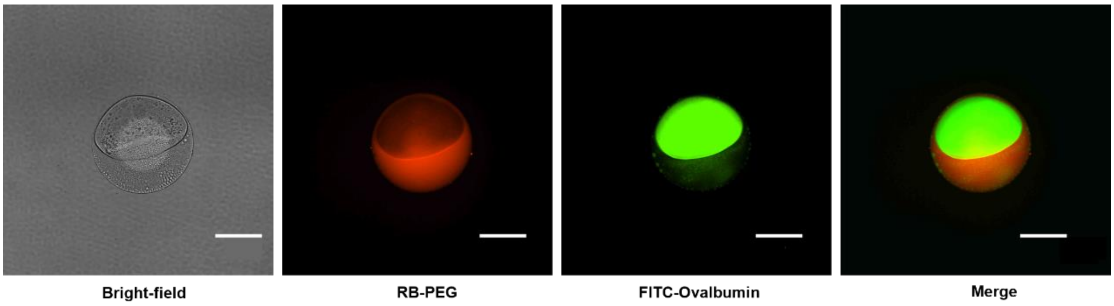

Supplementary Figure 11. Morphology of an evaporating ATPS droplet on an oil-wetted substrate resulting from segregative LLPS between PEO and dextran. The droplet contains 3.0 wt % dextran and 1.0 wt % PEO with a volume ratio of 1:1. Neither Pluronic F-68 nor RSN-0749 is added. Scale bars are 200 μm.

Supplementary References

[1] H. Kim, F. Boulogne, E. Um, I. Jacobi, E. Button, H.A. Stone, Phys. Rev. Lett. 116 (2016) 124501.

[2] W. Guo, A.B. Kinghorn, Y. Zhang, Q. Li, A.D. Poonam, J.A. Tanner, H.C. Shum, Nat. Commun. 12 (2021) 3194.
